# Substance Use and Chronic Pain Management: Understanding the Learning Needs of Primary Care Clinicians Through Project ECHO

**DOI:** 10.3390/healthcare13080873

**Published:** 2025-04-11

**Authors:** Joanna G. Katzman, Brandon J. Warrick, Mikiko Takeda, Snehal Bhatt, Radhika P. Grandhe, Vanessa Jacobsohn, Laura E. Tomedi

**Affiliations:** 1 Department of Neurosurgery, School of Medicine, Health Sciences Center, University of New Mexico, Albuquerque, NM 87106, USA; 2 Department of Emergency Medicine, University of New Mexico, Albuquerque, NM 87131, USA; brandonwarrick@salud.unm.edu; 3 Department of Pharmacy Practice and Administrative Sciences, College of Pharmacy, University of New Mexico, Albuquerque, NM 87106, USA; myamada@salud.unm.edu; 4 Department of Psychiatry and Behavioral Sciences, University of New Mexico, Albuquerque, NM 87106, USA; sbhatt@salud.unm.edu (S.B.); vjacobsohn@salud.unm.edu (V.J.); 5 Department of Anesthesiology, University of New Mexico, Albuquerque, NM 87131, USA; rgrandhe@salud.unm.edu; 6 College of Population Health, Health Science Center, University of New Mexico, Albuquerque, NM 87131, USA; ltomedi@salud.unm.edu

**Keywords:** acute pain, chronic pain, opioid management

## Abstract

**Background:** In 2022, more than 107,000 people died in the US from an opioid overdose. The Opioid Rapid Response and Pain (ORRP) ECHO was developed to educate primary care clinicians on best practices in pain and substance use management consistent with the 2016 Guideline for Prescribing Opioids for Chronic Pain. **Methods:** Six 1 h virtual sessions consisting of didactics and case discussions focusing on pain and substance use were delivered by a multidisciplinary hub team to four diverse US regions. The authors utilized qualitative analyses, including a modified Delphi Technique and thematic analysis, to assess participant questions during the sessions, focus groups with participants, and post-session survey responses for ORRP ECHO training between 14 October 2021 and 15 November 2022. **Results:** One hundred and eighteen primary care clinicians performed 627 chat responses and were eligible to receive 648 continuing education unit credits. The majority of chat questions were related to Patient-Centered Care (28%, 170 total responses) and Knowledge (27%, 178 total responses). The focus groups revealed five core themes: (1) there was a clear need for the ECHO trainings, (2) the program fostered a unique and supportive community, (3) the content was applicable, (4) the administration of the program was effective, and (5) it had a meaningful impact on practice. The participants who completed the post-session surveys reported that they would apply their knowledge (range across cohorts: 85.7% to 100.0% of survey respondents agreed or strongly agreed) and communication skills gained (range: 71.4% to 98.6%). **Conclusions:** Through didactics and interactive case discussions, the ECHO model holds promise as a useful training model to support the appropriate use of clinical practice guidelines by informing individualized, patient-centered care and clinical judgment.

## 1. Introduction

The number of Americans dying from drug overdoses continues to rise, with 107,941 deaths recorded in 2022, the majority of these involving synthetic opioids such as illegally made fentanyl, often combined with other substances such as methamphetamine and alcohol [1,2]. While prescription opioids play a vital role in pain management, their misuse can lead to opioid use disorder and overdose [3,4,5]. Chronic pain affects approximately one in five U.S. adults, and many individuals with substance use disorders have a history of inadequately treated pain [6,7]. This underscores the critical need for effective clinician training on opioid prescribing and pain management [8].

The Centers for Disease Control and Prevention (CDC) released opioid prescribing guidelines in 2016, updated in 2022, to help clinicians balance the benefits and risks of opioid therapy [9,10]. However, the misinterpretation or inflexible application of these guidelines can harm patients, highlighting the need for education that supports evidence-based, patient-centered care [11].

Project ECHO (Extension for Community Healthcare Outcomes), developed in 2003 at the University of New Mexico (UNM), is a virtual telementoring program designed to democratize medical knowledge and improve care for underserved populations [12]. ECHO uses a hub-and-spoke model where expert teams (hubs) provide guidance to participating clinicians (spokes) through case-based learning and collaborative discussion. This “all-teach, all-learn” approach builds communities of practice and enhances clinician self-efficacy [13]. Over the years, ECHO programs have successfully translated clinical guidelines into practice, improving outcomes in areas such as chronic pain, substance use, and infectious diseases [14,15,16].

There are now 1318 ECHO hubs in 208 countries, with thousands of medical, public health, and educational programs available [15]. As of June 2024, Project ECHO has provided programming for over 126 million attendances by learners [15].

The Opioid Rapid Response and Pain (ORRP) ECHO, modeled on prior successful ECHO pain programs, was developed in collaboration with the CDC to train primary care clinicians on best practices in pain and substance use management based on the 2016 CDC Guidelines [9]. The ORRP ECHO targeted clinicians in communities experiencing disruptions in pain or addiction care due to the abrupt loss of a prescribing clinician, a situation that places displaced patients at a high risk of unintentional overdose [17].

### Study Aim

This study aimed to evaluate whether the ORRP ECHO improved clinician knowledge, self-efficacy, and behavior related to the 2016 CDC Guidelines for Prescribing Opioids for Chronic Pain [9]. By analyzing Zoom chat logs, focus group evaluations, and post-session survey data, we sought to understand how Project ECHO’s telementoring model supports guideline implementation in primary care settings.

## 2. Methods

### 2.1. ECHO Model and Design

The Opioid Rapid Response and Pain (ORRP) ECHO was developed to educate civilian primary care clinicians (PCCs) on best practices in pain and substance use management, consistent with the 2016 CDC Guidelines [9]. The more recent 2022 CDC Guidelines were not utilized because the ORRP program was just completed before the newer guidelines were published [9,10]. The ORRP ECHO program was modeled after the successful Project ECHO USPHS (US Public Health Service) and CDC collaboration, whereby USPHS officers were trained by the Project ECHO pain team [18].

The ORRP ECHO hub team of subject matter experts (SMEs) included the following specialties: neurology, anesthesia/pain management, addiction psychiatry, family medicine/addiction, and emergency medicine/medical toxicology/addiction. The SMEs delivered 6 weeks of 1 h virtual pain and substance use telementoring sessions to primary care clinicians in communities in which a specialty care doctor had just lost their pain or addiction license. The goal was to educate PCCs in 4 U.S. regions: the Southwest, Northeast, Midwest, and Appalachia. (The ORRP ECHO series was given twice to the Appalachia region because of their scheduling difficulties). The clinical sites in each of the geographic regions were based upon the urgent clinical need arising in a particular jurisdiction. Urgent situations included a pain or addiction specialist losing their controlled substance prescribing license due to regulatory or legal action or a clinician abruptly leaving their practice. As part of their agreement to participate in the ORRP ECHO trainings, these clinicians agreed to accept these soon-to-be displaced patients. In addition, the participating clinicians had support from their various clinics’ medical and administrative directors, who realized the value of this protected educational experience for their clinicians. Each session included an evidence-based didactic, presented by a hub team member, a de-identified and Health Insurance Portability and Accountability Act (HIPAA)-compliant case-based learning component from a participating clinician, and time for discussion. Didactic topics included the following: Epidemics of Chronic Pain; Substance Use Disorders and Suicide; How to Safely Care for Patients with Chronic Pain and Substance Use Disorder; Non-Opioid Pharmacotherapy for Chronic Pain; How to Taper Opioid Analgesics; How to Taper Benzodiazepines; Medications for Opioid Use Disorder. See Table 1.

During the ECHO sessions, participants were encouraged to send messages via the chat feature when they had questions, comments, or information to share related to opioid and/or pain management. All chat messages were saved by Project ECHO and distributed to clinician investigators for review.

### 2.2. Participants

Participants registered for the cohort using a Redcap survey and reported their credentials and organization [19]. Credentials were categorized as follows: medical doctors (MDs); physician assistants (PAs); nurse practitioners (NPs); nurses or nurse midwives; social workers; pharmacists; and others. As well as being grouped by geographical location, organizations were grouped by place of work into the following categories: federally qualified health centers, non-profit hospitals, academic health centers, federal agencies, and other clinics or agencies (e.g., health department, private clinic, etc.). We conducted descriptive statistics (e.g., frequencies and means) analysis to explore the sociodemographic characteristics of participants by region.

### 2.3. Zoom Chat Analysis

To develop the Zoom chat category codes, one of the hub team clinicians evaluated the chat messages and generated preliminary categories based on the frequency of topics discussed. This was followed by a hub team discussion, in which the categories were further refined. Six focus areas were used for coding: Ethics, Debunking of Misinformation, Flexibility/Boundaries, Knowledge, Patient-Centered Care, and Resources. In addition, an “Other” category was created for all questions unrelated to the course content. Chat messages could be classified in more than one category. See Table 2.

The categorization of individual chat messages was performed using a modified Delphi Technique [20]. Each pain-specialized clinician was paired with an addiction-specialized clinician to evaluate the chat responses. The first-round chats were independently reviewed by each pair of physicians, followed by a team discussion to identify any disagreements and then a discussion to reach a consensus. Three rounds of review and discussions were performed to categorize each chat message accurately for study purposes.

Once a consensus was reached, the numbers of chats in the six focus areas from each cohort were counted and compared. The top three questions or comments from each chat category were then identified. See Table 3.

### 2.4. Focus Groups

We conducted four focus groups between October 2022 and May 2023 using a standardized script to evaluate the ECHO program. Focus groups were announced during the final session of each six-week cohort training, with CDC and hub staff excused to ensure candid responses. Conducted over Zoom approximately one week after each cohort ended, the sessions lasted 30–60 min, were moderated by a trained evaluator, and included 1–5 participants in the following groups: Appalachia cohort #1 (5 participants on 12 October 2022), Midwest (1 participant on 20 October 2022), Appalachia cohort #2 (3 participants on 22 November 2022), and Northeast (4 participants on 19 May 2023). We did not conduct a focus group with the Southwest cohort because focus groups were implemented after this cohort’s training was completed. All ECHO program participants were invited to be involved in the focus groups. Demographic characteristics were not collected for focus group participants. Discussions were recorded, transcribed, and coded independently by two evaluators using NVivo 14 [21]. Inductive coding was employed, with codes developed during text review and finalized through consensus. Themes were identified iteratively from the coded data. Although one focus group had only one participant, it was analyzed alongside the others for consistency. Participants were asked about the anticipated changes to their practice, the utility of the ECHO sessions, and potential program improvements.

### 2.5. Post-Session Survey

After each session, an online, voluntary post-session survey was provided to participants using REDCap [19]. The survey was tied to participants receiving continuing medical education (CME) or continuing education unit (CEU) credits and asked participants about the administration of the session (results not included here) and the short-term impact of the session using their agreement (strongly disagree, disagree, neutral, agree, and strongly agree) with the following statements: “I intend to apply the knowledge and/or skills I have acquired from this activity to my work when in a team environment”, “I am better able to communicate with other members of a multidisciplinary team as a result of what I learned in this activity”, and “I am better able to discuss how teamwork can contribute to continuous and reliable patient care”. The responses were aggregated across sessions in the cohorts; therefore, participants were included multiple times if they responded to the surveys for multiple sessions (i.e., the reported numbers are numbers of surveys, not participants).

This evaluation protocol was reviewed and approved by the University of New Mexico Health Sciences Center Institutional Review Board (#04-341).

## 3. Results

### 3.1. Demographics

A total of 118 clinicians participated and were eligible to receive up to 708 no-cost continuing medical education (CME) or continuing education unit (CEU) credits. This included 35% of clinicians in the Southwest region, 23% in the Northeast region, 14% in the Midwest region, and 28% in the Appalachia region. The majority of the PCCs worked as physicians, nurse practitioners, or physician assistants and worked in federally qualified health care centers (FQHCC). The Appalachia region had the most diverse group of clinicians, because of their larger representation of nurses and pharmacists, compared to the other regions. In addition, the Midwest region had several primary care clinicians with previous addiction training. See Table 4.

### 3.2. Chat Analysis

Patient-Centered Care and Knowledge were the two largest chat categories, representing 28% and 27% of the total chat questions, respectively. See Table 5A. Debunking/Misinformation was the smallest category, with less than 2% of the questions. The “Other” category comprised 24% of the chat questions. Examples of chats in the “Other” category include comments/questions unrelated to the didactic content. These included questions for the Project ECHO staff and personal chats to other participants. See Table 5 (Table 5A,B).

### 3.3. Chat Categories

#### 3.3.1. Patient-Centered Care

“How do you know if the patient is willing to taper his/[her] benzodiazepines?”

“What is the underlying source of his/her anxiety? Maybe he/[she] would benefit from a behavioral health referral”.

Patient-Centered Care was the most common chat category, with 178 chat instances. The most frequently asked patient-centered care chat questions and comments focused on opioid tapering best practices. See Table 5A. There were repeated discussions about strategies to manage patients for whom different tapering methods were not the best fit.

The nextmostcommon type of Patient-Centered Care chat questions were related to safety and efficacy when utilizing non-opioid pharmacotherapy and non-pharmacologic treatments for chronic pain. The third most common topics regarded the management of co-existing mental health conditions of anxiety, post-traumatic stress disorder (PTSD), and substance use disorders. Illustrative chat conversations included the following topics: how to taper co-prescribed opioids and benzodiazepines and how to care for patients with chronic pain without using prescription opioids. Participants were particularly interested in knowing about non-benzodiazepine alternatives to managing anxiety, best practices for benzodiazepine tapering, the management of substance use disorders, the use of buprenorphine for pain management, and buprenorphine micro-dosing strategies.

#### 3.3.2. Knowledge

“Is high MME alone enough to taper someone who is on opioids?”

Regarding chat questions and comments related to Knowledge, the participating clinicians were most interested in safety issues when tapering high-dosage opioids, the importance of treating substance use disorders co-occurring with chronic pain, and whether or not an acute opioid overdose may trigger the immediate discontinuation of an opioid prescription and/or necessitate a non-opioid medication.

#### 3.3.3. Ethics

“I see many substance use patients who do not want to get treatment unless there is a court order. Not sure what to do”.

The Ethics chat questions and comments focused on the following issues: whether or not to continue opioid analgesics or begin an opioid taper, how a clinician can be both responsive to a patient’s concerns while expressing their own safety concerns, and how to address medication compliance or possible diversion.

#### 3.3.4. Debunking of Misinformation

“We see numerous patients here who have this same benzodiazepine-opioid combo and have been on the combo for years. Most are very possessive of these meds and hate attempts to taper”.

There were only 10 chat comments and/or questions related to the Debunking of Misinformation, but the authors felt it important to include this category to distinguish it from the Knowledge and “Other” categories. Some other illustrative chat quotes included the following: “Isn’t methadone ‘feeding’ the addiction just like all the other opioids?”. These chat comments/questions illustrated topics that needed debunking from the hub team SMEs in order to correct the misinformation from the PCCs joining the ORRP ECHO session.

#### 3.3.5. Flexibility/Boundaries

“I think that the toughest patients to taper are the folks who have tried a lot of different things without success—in that case I find it really difficult to convince them [the patients] when I am simply taking away medication. The taper will be most successful if it comes from the patient, but I need to go slow”.

The most common Flexibility/Boundaries chat questions and comments were related to when to taper (or not) opioids taken by patients with severe chronic pain who have benefitted from opioid analgesics but have medical co-morbidities putting them at a high risk. Other topics included (1) appropriate clinical options when a toxicology report indicates that the patient is not taking the prescribed opioid analgesic and (2) how to treat patients who are functioning well on small amounts of opioids and benzodiazepines concurrently.

#### 3.3.6. Resources

“What are some of the best non-pharmacological resources for patients with insomnia?”

The three most common chat questions and comments related to Resources were (1) non-pharmacological care, including physical rehabilitation and behavioral medicine, (2) the importance of discussing and referring patients to pain, substance use, and behavioral health specialists, and (3) the use of validated screening tools.

### 3.4. Focus Group

The ORRP ECHO focus groups revealed five core themes: (1) there was a clear need for the ECHO trainings, (2) the program fostered a unique and supportive community, (3) the content was highly applicable to the participants’ work, (4) the administration of the program was effective, and (5) the program had a meaningful impact on practice. Participants highlighted the need for the ECHO program, particularly in addressing the challenges of managing patients on risky opioid prescriptions and tailoring care to rural settings. One participant explained, “What [helped] me the most is trying to identify patients that might be willing to try to have some non-opioid alternatives to what they’re on”. The program’s community aspect was also widely appreciated, with interdisciplinary participation allowing participants to learn from varied perspectives. One participant noted, “You just get to learn different ways of thinking about, or focusing on, an issue. To me, that’s extremely helpful and very rare”. Additionally, participants praised the practical content and the knowledgeable hub team, whose guidance provided “special tips and tricks, little pearls”. The suggestions for improvement included increased recruitment, better timing, and more inpatient or emergency care content. Overall, the ECHO program effectively addressed critical clinical needs and provided participants with valuable tools and insights for their practice.

### 3.5. Post-Session Survey

The response rates to the post-session surveys ranged from 15% to 33% [Southwest (n = 71 surveys, 33% response rate), Northeast (n = 54 surveys, 27% response rate), Midwest (n = 14 surveys, 15% response rate), and Appalachia (n = 55 surveys, 30% response rate)]. The response rates are the number of surveys over the number of attendees, not unique participants, because these numbers were aggregated across the cohort. For the question: “I intend to apply the knowledge and/or skills I have acquired from this activity to my work when in a team environment”, 71 (100%) survey responses from the Southwest cohort, 52 (96.3%) survey responses from the Northeast cohort, 12 (85.7%) survey responses from the Midwest cohort, and 50 (90.9%) survey responses from the Appalachia cohort said that they agreed or strongly agreed with this statement. For the question: “I am better able to communicate with other members of a multidisciplinary team as a result of what I learned in this activity”, 70 (98.6%) of the Southwest, 47 (87.0%) of the Northeast, 10 (71.4%) of the Midwest, and 48 (87.3%) of the Appalachia survey respondents said they agreed or strongly agreed. Lastly, a total of 60 (97.2%) of the Southwest, 48 (87.3%) of the Northeast, 11 (78.6%) of the Midwest, and 48 (87.3%) of the Appalachia survey participants said that they agreed or strongly agreed with the statement “I am better able to discuss how teamwork can contribute to continuous and reliable patient care”.

## 4. Discussion

The ORRP ECHO trainings demonstrated that PCCs and other frontline health professionals were able to successfully engage with UNM’s Project ECHO for six 1 h trainings and learn evidence-based guidelines related to chronic pain and opioid management. This was supported by chat analysis, focus group surveys, and weekly post-session surveys. This 6-week, 1 h/per week program proved to be successful for busy PCCs volunteering for nuanced training in pain and opioid management. In 2019, Project ECHO collaborated with the US Public Health Service and the Centers for Disease Control and Prevention (CDC) to train 163 USPHS health professionals in best practices of pain management and opioid prescribing over two 16-week series [18]. During this time, formal didactics and case-based learning—using the 2016 CDC Guidelines for Prescribing for Chronic Pain—were provided to the clinicians, who significantly improved their knowledge, self-efficacy, and changed their practice behavior [11,12]. This USPHS series (described above) lasted for 16 weeks, as compared to the much-abbreviated 6-week course in the current study [18].

The Zoom chat analysis, focus groups, and post-session surveys in this study demonstrate that the participants benefit greatly from attending these six ORRP ECHO sessions, increasing their knowledge and confidence and even positively impacting their practice behavior after learning about the 2016 CDC Guideline for Prescribing Opioids for Chronic Pain during the ECHO sessions [9]. The aims of this study—the improvement of knowledge and self-efficacy, along with behavioral change—were all accomplished.

### 4.1. Project ECHO Telementoring Translates Guidelines into Practice

Since Project ECHO began, many ECHO programs have developed a goal to educate health professionals (usually primary care clinicians) on evidence-based practices [22]. During the global COVID-19 pandemic, Project ECHO began to provide virtual just-in-time COVID-19 (and other) telementoring consultations [23]. Some ECHO programs also developed COVID-19 best practice programs with the aim of delivering evidence-based and evidence-guided directives from the CDC or the Infectious Disease Society of America (ISDA) so that practitioners could learn the best treatment options for their patients with COVID-19 [24,25].

There are other ECHO programs whose primary mission is translating guidelines into practice. These include the Texas HPV ECHO, which trains Community Health Workers to understand the importance of the HPV vaccine in the prevention of cervical cancer so that they can then educate their clients [26]. Additionally, the Sickle Cell ECHO in the Midwest uses Project ECHO to successfully disseminate Sickle Cell guideline information about evidence-based best practices for medical management to clinicians [27].

Based on this study’s chat analysis, the 118 PCCs completing the 6 h telementoring ORRP ECHO training were primarily interested in topics related to Patient-Centered Care and Knowledge. However, the list of identified chat categories (Ethics, Flexibility/Boundaries, Knowledge, Misinformation/Debunking, Patient-Centered Care, and Resources) likely reflects gaps in understanding that PCCs have across the nation. These themes also reflect the complexity of PCCs’ needs when providing opioid care. While prescribing information, non-opioid alternatives, and tapering algorithms are important, primary care clinicians also need information on, and ongoing support for, addressing the ethical considerations and navigating patients’ trepidation about reducing or altering their medications. The focus group results also indicated that the ECHO model created a supportive environment for PCCs to seek support for these more complex issues. Future research could better understand these knowledge deficits and provide practical applications in assisting PCCs who are inheriting patients on high doses of opioids from a previous clinician, whether due to the loss of their license or an abrupt move out of the region.

This study illustrates the real-world application of the 2016 CDC Guideline for Prescribing Opioids for Chronic Pain, along with the need for additional pain and substance use training [9]. Moreover, this demonstrates that, when chronic pain and opioid management guidelines need to be updated and/or initiated for the first time, the ECHO model may be used as a successful capacity-building format.

The ECHO model provides case-based learning combined with didactics and discussion between the SMEs and the clinician participants, providing just-in-time, individualized recommendations for each unique patient that is presented during the ORRP ECHO trainings [28]. The Project ECHO ORRP hub team continues to work with the CDC in new jurisdictions across the country to promote guideline management, and is now using the 2022 CDC Guideline for Prescribing Opioids for Chronic Pain [10].

### 4.2. Limitations

The authors are cognizant that just as their telementoring trainings finished the 2022 CDC Clinical Practice Guideline was officially published [10]. The authors believe that the ORRP ECHO trainings remained evidence-based and just as clinically valid for the clinicians who were trained—even though the 2022 CDC Practice Guidelines had not been published yet [10].

Although participants were chosen from several different practice locations, the 118 participants may not reflect the opinions of PCCs nationally. Also, given the format, some participants may have been more comfortable than others to ask questions using the Zoom chat function, and this may have resulted in reporting bias.

While the Zoom chat analysis portion of this study offers a unique insight into the needs of PCCs providing frontline opioid care, there are limitations that should be considered when reviewing this analysis. Although the authors conducted two independent expert reviews of each line of text to reduce analyst bias, qualitative analysis is inherently subjective. The focus group and post-session surveys, however, complement the Zoom chat results.

## 5. Conclusions

In conclusion, the ORRP ECHO sessions provided successful, time-limited, evidence-based telementoring opportunities for clinicians desiring to learn pain and substance use guideline management. Given the alarming rise of illegal fentanyl, PCCs remain on the frontlines of this public health emergency. The results described in this article illuminate the complex nature of treating chronic pain and substance use disorders and the need for practical, time-limited iterative trainings. Virtual learning models, such as Project ECHO, can be replicated given this capacity-building learning model and can be used for pain and substance use best practice trainings, providing evidence-based guidelines, case-based learning, and a community of practice.

## Figures and Tables

**Table 1:** Opioid Rapid Response and Pain ECHO Curriculum—14 October 2021 to 15 November 2022.

Week	Title	Objective 1	Objective 2	Objective 3
1	Epidemics of Chronic Pain, Substance Use Disorders and Suicide in the United States	To understand the current epidemiologic prevalence of chronic pain and substance use disorders.	To describe the interactions among chronic pain, substance use disorders, mental health, and suicide.	To recognize that social determinants of health and stigma are associated with each of these disorders.
2	How to Safely Care for Patients with Chronic Pain and Substance Use Disorder	To recognize the additive risks of prescription opioids with benzodiazepines and alcohol.	To appreciate that treatment should NOT be initiated with a long-acting opioid formulation.	To understand the best screening tools for depression and suicide.
3	Non-Opioid Pharmacotherapy for Chronic Pain	To name the various categories of non-opiate pain medications,	To identify the indications of a prototypical medication from each category of non-opioid pain medications.	To become familiar with the contraindications and side effect profiles of various classes of non-opioid pain medications.
4	How to Taper Opioid Analgesics	To appreciate some common reasons for opioid tapering.	To be able to communicate effectively with a patient the reasons for and goals of the taper.	To learn the basic steps of carrying out opioid tapers.
5	How to Taper Benzodiazepines	To be able to cite the risks of long-term benzodiazepine use.	To learn the basics of carrying out benzodiazepine detoxifications.	To learn the best practices of effective communication with patients who need to undergo benzodiazepine detoxification.
6	Medications for Opioid Use Disorders	To appreciate that detoxification alone is not an effective treatment for opioid use disorders.	To appreciate that substance use disorders are chronic illnesses.	To learn that maintenance treatment with an FDA-approved medication is the standard of care for patients with opioid use disorders.

**Table 2:** Chat Categories: Opioid Rapid Response and Pain ECHO Curriculum—14 October 2021 to 15 November 2022.

Categories	Description/Includes
Boundaries/Flexibility	Sometimes clinical patient care requires personalized approaches to treatment. Not every person can have the same situation; therefore, there must be some flexibility in which clinicians can make patient-centered management choices.
Debunking of Misinformation	It is critical in clinical medicine to disprove false information in order to provide optimal quality patient care. The most recent evidence-based and evidence-guided information was always provided in every ECHO session.
Ethics	The risks/benefits of medication tapers, the importance of never abandoning a patient, secondary gain vs. belief in a patient, the moral imperative of treating opioid use disorder and chronic pain concurrently, the conflict when a clinician’s beliefs are not synonymous with the patient’s best interests or when self-disclosure is appropriate, and the implicit bias of clinicians/clinician stigma.
Knowledge	This included any clinician question pertaining to patient care. For instance, screening tools, how to interpret urine toxicology screens, the best peri-operative management of pain, the optimal treatment of chronic migraine, etc.
Patient-Centered Care	This included empathy, compassionate listening, a whole-person view of the patient, active listening, trauma-informed care, protective factors, resilience factors, spirituality, and social supports
Resources	This included barriers to transportation, rehabilitation services (including pool availability), Medicaid coverage, behavioral health, housing, social work consults, self-care programs, provider self-care programs, and primary care/specialty access.

**Table 3:** Results of “Top Three Chat Questions and Comments” in each category. Opioid Rapid Response and Pain ECHO Curriculum—14 October 2021 to 15 November 2022.

Category	Question/Comment 1	Question/Comment 2	Question/Comment 3
Boundaries/ Flexibility	How do I approach a patient who has benefitted from opioid analgesics due to severe pain yet has medical co-morbidities?	If the opioid analgesic of concern is not in the confirmation toxicology, how do I discuss my concerns with the patient regarding management and opioid taper?	If I inherit a patient who seems to be doing well but is taking small amounts of both opioid analgesics and benzodiazepines, how do I approach this patient to discuss my concerns?
Debunking of Misinformation	Patients “hate” to taper their opioids.	How do I educate patients on the risk of opioid overdose when taking both opioids and benzodiazepines?	Having a patient move from opioids for pain to methadone is just “feeding” the addiction.
Ethics	When assessing a patient regarding continuing their opioid analgesic therapy (and at what dose), a holistic risk/benefit analysis is needed.	How do I communicate our concerns and also be responsive to the patient’s concerns?	How best do I address concerns about diversion and lack of compliance?
Knowledge	What do studies show about safety of tapering high dose opioids, weaning opioids or switching to another medication opioid, such as buprenorphine?	Can you explain the difference between substance use disorder, misuse and diversion?	If a patient has an acute opioid overdose or is diverting medication, I know this should trigger immediately stopping opioid prescription/provide alternative. Are there other instances when I should abruptly stop the opioid?
Patient-Centered Care	How do I use suboxone for opioid use disorder? (optimal dosing, how do I start, etc.)	How do I use buprenorphine for pain management and what are the benefits compared to pure mu opioid agonist?	How do I taper benzodiazepines?
Resources	Non-pharmacological chronic pain resources include: PT, exercise, behavioral medicine and phone apps.	Validated pain and mental health tools include CBTi, COMM, PHQ-9, ACE score *.	Referrals to other specialists for further work-up: sleep medicine, pain medicine, and social work/care coordination when indicated.

* CBTi = Cognitive Behavior Therapy for Insomnia; COMM = Current Opioid Misuse Measure; PHQ-9 = Patient Health Questionnaire for Depression; ACE Score = Adverse Childhood Experiences Score.

**Table 4:** Demographics—Opioid Rapid Response and Pain ECHO Curriculum—14 October 2021 to 15 November 2022.

	Southwest	Northeast	Midwest	Appalachia	Total
Dates	14 October–18 November 2021	7 April–12 May 2022	1 September–13 October 2022	21 August–5 October 2022 and 11 October–15 November 2022	
Total number of participants	41	27	17	33	118
Profession, N (%)					
MD/PA/NP	28 (68.3)	22 (81.5)	9 (52.9)	9 (27.3)	68
Nurse/nurse midwife	4 (9.8)	1 (3.7)	1 (5.9)	8 (24.2)	14
Social worker	2 (4.9)	1 (3.7)	1 (5.9)	1 (3.0)	5
Pharmacist	0 (0.0)	0 (0.0)	1 (5.9)	8 (24.2)	9
Other	2 (4.9)	2 (7.4)	4 (23.5)	4 (12.1)	12
Missing	5 (12.2)	1 (3.7)	1 (5.9)	3 (9.1)	10
Place of work, N (%)					
Federally qualified health clinic	28 (68.3)	19 (70.4)	13 (76.5)	8 (24.2)	68
Non-profit hospital	0 (0.0)	0 (0.0)	0 (0.0)	14 (42.4)	14
Academic health center	3 (7.3)	1 (3.7)	1 (5.9)	3 (9.1)	8
Federal agency	4 (9.8)	3 (11.1)	3 (17.7)	3 (9.1)	13
Other clinic or agency	2 (4.9)	2 (7.4)	0 (0.0)	5 (15.2)	9
Missing	4 (9.8)	2 (7.4)	0 (0.0)	0 (0.0)	6

MD = medical doctor; PA = physician assistant; NP = nurse practitioner.

**Table 5:** Chat counts, total and by region: Opioid Rapid Response and Pain ECHO Curriculum—14 October 2021 to 15 November 2022. (**A**): Total counts. (**B**): Counts by region.

A								
	Boundary	Debunking	Ethics	Knowledge	PCC *	Resources	Others	Total
%	3.8	1.7	5.7	27.2	28.4	9.2	24	100
count	24.1	10.5	35.9	170.4	177.9	57.8	150.3	627
**B**								
	**# of Chats**	**Boundary (%)**	**Debunking (%)**	**Ethics** **(%)**	**Knowledge (%)**	PCC * (%)	**Resources (%)**	**Others (%)**
Southwest	204	2.2	0.5	7.4	30.9	26.9	8.5	23.5
Northeast	223	5.6	3.6	6.3	24.7	25.3	11.2	23.3
Appalachia	119	6	1.5	5.3	33.3	39.5	10.5	22.8
Midwest	81	1.2	0	1.9	23.5	33.3	6.2	34

* Patient-Centered Care.

## Data Availability

The data and materials from this manuscript are not publicly available.
